# Limiting treatment plan complexity by applying a novel commercial tool

**DOI:** 10.1002/acm2.12908

**Published:** 2020-05-21

**Authors:** Alessandro Scaggion, Marco Fusella, Giancarmelo Agnello, Andrea Bettinelli, Nicola Pivato, Antonella Roggio, Marco A. Rossato, Matteo Sepulcri, Marta Paiusco

**Affiliations:** ^1^ Medical Physics Department Veneto Institute of Oncology IOV‐IRCCS Padova Italy; ^2^ Radiation Oncology Department Veneto Institute of Oncology IOV‐IRCCS Padova Italy

**Keywords:** aperture shape controller, complexity metrics, MLC, plan complexity, plan quality metric, treatment planning

## Abstract

**Purpose:**

A recently introduced commercial tool is tested to assess whether it is able to reduce the complexity of a treatment plan and improve deliverability without compromising overall quality.

**Methods:**

Ten prostate and ten oropharynx plans of previously treated patients were reoptimized using the aperture shape controller (ASC) tool recently introduced in Eclipse TPS (Varian Medical Systems, Palo Alto, CA). The performance of ASC was assessed in terms of the overall plan quality using a plan quality metric, the reduction in plan complexity through the analysis of 14 of the most common plan complexity metrics, and the change in plan deliverability through 3D dosimetric measurements. Similarly, plans optimized limiting the total number of delivered monitor units was assessed and compared. The two strategies were also combined to assess their potential combination.

**Results:**

The plans optimized by exploiting the ASC generally show a reduced number of total Monitor Units, a more constant gantry rotation and a MLC modulation characterized by larger and less complicated shapes with leaves traveling shorter overall lengths.

**Conclusions:**

This first experience suggests that the ASC is an effective tool to reduce the unnecessary complexity of a plan. This turns into an increased plan deliverability with no loss of plan quality.

## 1 Introduction

Volumetric modulated arc therapy (VMAT) is nowadays the standard treatment technique in high‐quality radiation therapy delivered with clinical LINACs. The rapid dose fall‐off outside target boundaries grants highly conformed dose distributions and near‐optimal sparing of surrounding critical structures. Volumetric modulated arc therapy has generally replaced intensity‐modulated radiation therapy (IMRT) because of the shorter treatment time obtained through the simultaneous variation of gantry speed, dose rate and MLC position.^1^, ^2^ Moreover, VMAT tends to use fewer monitor units (MU) per fraction than IMRT and thus reduces the burden of second malignancies.^3^, ^4^, ^5^ However, the potential downsides of VMAT include the augmented low dose radiation to surrounding normal tissue^2^ and the large complexity of delivery characterized by intensive MLC modulation, that is, a large amount of small MLC subfields. Such complexity might undermine dose calculation correctness and increase the uncertainty of the dose verification process.^6^, ^7^, ^8^, ^9^

These drawbacks of VMAT have led to the proliferation of studies dedicated to the measurement of VMAT plan complexity and related plan quality and deliverability. It has been shown that unnecessary amount of complexity negatively affects the accuracy of both dose calculations and treatment delivery, in particular for TPS based on simple MLC physics models.^10^, ^11^, ^12^, ^13^, ^14^, ^15^, ^16^, ^17^, ^18^, ^19^

Part of these studies were dedicated to the exploration of strategies to limit this complexity. In this respect, many strategies have been proposed to limit plan complexity, primarily reducing the delivered MU or directly simplifying the shape of the MLC during optimization.^9^, ^11^, ^20^, ^21^, ^22^, ^23^, ^24^ To limit VMAT complexity, a similar strategy has recently been introduced in v.15.5 of the Eclipse TPS (Varian Medical Systems, Palo Alto, CA). The aperture shape controller (ASC) is a new component in the leaf sequencer for VMAT in the photon optimizer (PO) algorithm that tends to increase the size and decrease the complexity of the MLC aperture.^25^

In this study, a first experience with ASC is reported. The strength of ASC in limiting VMAT plan complexity has been tested and compared to the MU limiting strategy implemented in Eclipse since v.8.6. Ten prostate and ten oropharynx clinical plans were reoptimized following different strategies to limit plan complexity. Every new plan was compared to the clinical plan to assess whether it is possible to limit plan complexity without compromising the overall clinical plan quality and if this reduction in complexity turns into an improved plan deliverability.

## 2 Methods and Materials

### 2.A Aperture shape controller

The aperture shape controller (ASC) is a new component in the leaf sequencer for VMAT in the PO algorithm. It was introduced in version 15.5 of the Eclipse TPS. The ASC favors apertures of minimal local curvature, which is measured as the positions of the tips of adjacent leaves that modulate the same spatially continuous target projection. ASC acts through a penalization term in the total cost function, the weight of which can be partially controlled by the user via a calculation option that can be set to five different values, ranging from very low to very high.^25^

### 2.B VMAT plans

Ten patients treated for low‐risk prostate cancer and ten patients treated for oropharyngeal cancer at our institution were retrospectively selected. The prostate patients were treated with 70 Gy in 28 fractions (2.5 Gy/fx) to the prostate gland only. The oropharynx patients were treated with 69.96 Gy to the tumor bed and 59.4 Gy to the nodal chains in 33 fractions (2.12 and 1.8 Gy/fx). All patients were treated using a TrueBeamSTx with high‐definition MLC (Varian Medical Systems, Palo Alto, CA, USA) and 6 MV energy.

According to manufacturer’s specifications, ASC attempts to join disconnected apertures defined by adjacent leaf pairs, which, in turn, may reduce the number of MUs delivered. On the other hand, it is also well known that limiting the delivered MU is an indirect way to limit MLC movement and modulation.^22^, ^26^ For this reason, three different strategies to limit MLC modulation were tested and compared: three plans were optimized by the same expert planner for each of the selected patients. For each patient the optimization was started with a set of constraints that was maintained practically unaltered for all the relative plans. The human interaction was minimal and devoted only to avoid possible major deviations. No postoptimization normalization was applied. A limit on the maximum Monitor Units was imposed, setting the MU objective to a total ratio of MUs/cGy equal to 3 with a fixed objective weight; this plan will be referred to as *MU limit.* ^16^ Another plan was optimized setting the ASC to very high penalty; this plan will be referred to as *ASC.* A further plan was optimized coupling the ASC penalty and the MU limit; it will be indicated as *ASC + MU limit*. The ASC was tested using the maximum available penalty to probe the limits of its capability. A detailed evaluation of which penalty level to use in a clinical environment is outside the scope of this study.

These plans were compared with the original clinical plans, which were optimized with no strategies to limit plan complexity. All plans were optimized with the Photon Optimizer v.15.5 and calculated with AcurosXB v.15.5 dose engine (Varian Medical Systems, Palo Alto, CA, USA). All plans used two full arcs with complementary collimator rotations with collimator angles ranging from 10 to 35 degrees.

### 2.C Plan quality assessment

To assess the overall plan quality and limit the subjectivity of judgment, the Plan Quality Metric (PQM) was adopted as a global measure of quality. PQM was first introduced by Nelms^27^ and is implemented in PlanIQ software (v2.1.1, Sun Nuclear Corp., Melbourne, FL). PQM is a user‐defined metric intended to compare the quality of treatment plans. It gathers the judgment of quality into a single number, mimicking that expressed by a clinical team. It is built through a list of submetrics, for example, DVH metrics, associated with a numerical scoring function which models as accurately as possible the judgment criteria of the clinicians. The PQM is the sum of the score obtained by each submetric and measures how much the plan adheres to a list of precompiled goals. The percentage PQM (PQM%), that is, the ratio of the achieved score to the maximum achievable thus represents a relative measure of plan soundness. In this study, two dedicated PQM algorithms were used to judge prostate and oropharynx plans. The algorithms were delineated in accordance with the clinicians and were inspired by published standards (RTOG0126, RTOG0522, RTOG0615, RTOG0619, RTOG0625, RTOG1016) and prior experience.^28^, ^29^ The detailed description can be found in the supplementary materials.

To pose further attention on dose conformity and dose spread, we also evaluated the variation of Conformity Index and the volume of healthy tissue receiving low doses. In particular, the following quantities were collected and compared: Conformation Number of 95% prescription dose to prostate PTV and to low dose oropharynx PTV^30^; the healthy tissue volume at 95%, 50%, and 30% of the prescription doses.

### 2.D Metrics of complexity

Following the existing literature, for each VMAT plan, we computed a number of complexity metrics related to the degree of modulation and plan deliverability. The computed metrics were selected with the intention of tracking all possible dose modulations occurring in a VMAT plan with a specific attention on MLC movement, given that ASC is devoted to limiting the unnecessary amount of MLC subfields in VMAT plans. Fourteen different metrics were considered. The complete list is given below, while for a systematic presentation, the reader is referred to the specific literature, or to.^19^, ^31^ RT‐plan DICOM files were imported into Matlab 2017a (the MathworksInc, Natick, MA, USA) and a dedicated routine was developed in‐house to compute: the total monitor units normalized to the prescription dose in cGy (***MU/cGy***), the leaf travel per arc length (***LTAL***), the mean Dose Rate Variation (***DRV***), the mean Gantry Speed Variation (***GSV***), the predicted delivery time (***dt***), the mean apertures area (***A***), the equivalent square field (***EFS***),^9^ the small aperture score (***SAS_10mm_***),^17^ the Edge Metric (***EM***),^11^ the Plan Irregularity (***PI***) and the Plan Modulation (***PM***),^16^ the Modulation Complexity Score (***MCS***)^12^ and its adaptation to VMAT (***VMCS***),^13^ and the total modulation index (***MI_t_***).^15^

### 2.E Pretreatment QA

Each of the plans was delivered in a unique and dedicated QA session on the ArcCHECK^TM^ detector array (Sun Nuclear Corporation, Melbourne, FL, USA) without making use of the PMMA CavityPlug^TM^. The reference 3D dose distribution for each plan was computed with AcurosXB v.15.5 on a homogenous virtual phantom, which was assigned 1.1836 g/cm^3^ density following manufacturer's recommendations.

Dosimetric comparisons were performed with SNC patient software (v. 6.7.2, Sun Nuclear Corporation, Melbourne, FL, USA), computing both local and global gamma evaluations. Gamma criteria of 2%/2 mm and 3%/3mm were used with 10% threshold of the maximum measured dose with both local (L) and global (G) normalization. The plans were considered deliverable if at least one of the following action levels of the gamma passing rate (GPR%) was met: 90% for 2%L/2 mm, 95% for 2%G/2 mm, 93% for 3%L/3 mm, and 97% for 3%G/3 mm. These cutoffs are based on the center’s experience and were set following the recommendation of AAPM report 119.^32^

### 2.F Data analysis

The clinical, complexity, and dosimetric outcome of the different optimization strategies were compared through two‐tailed paired Student’s *t*‐test, with a significance level of 0.05, once their normality was ensured using a Shapiro–Wilk test.

## 3 Results

### 3.A Plan quality

**Fig. 1:**
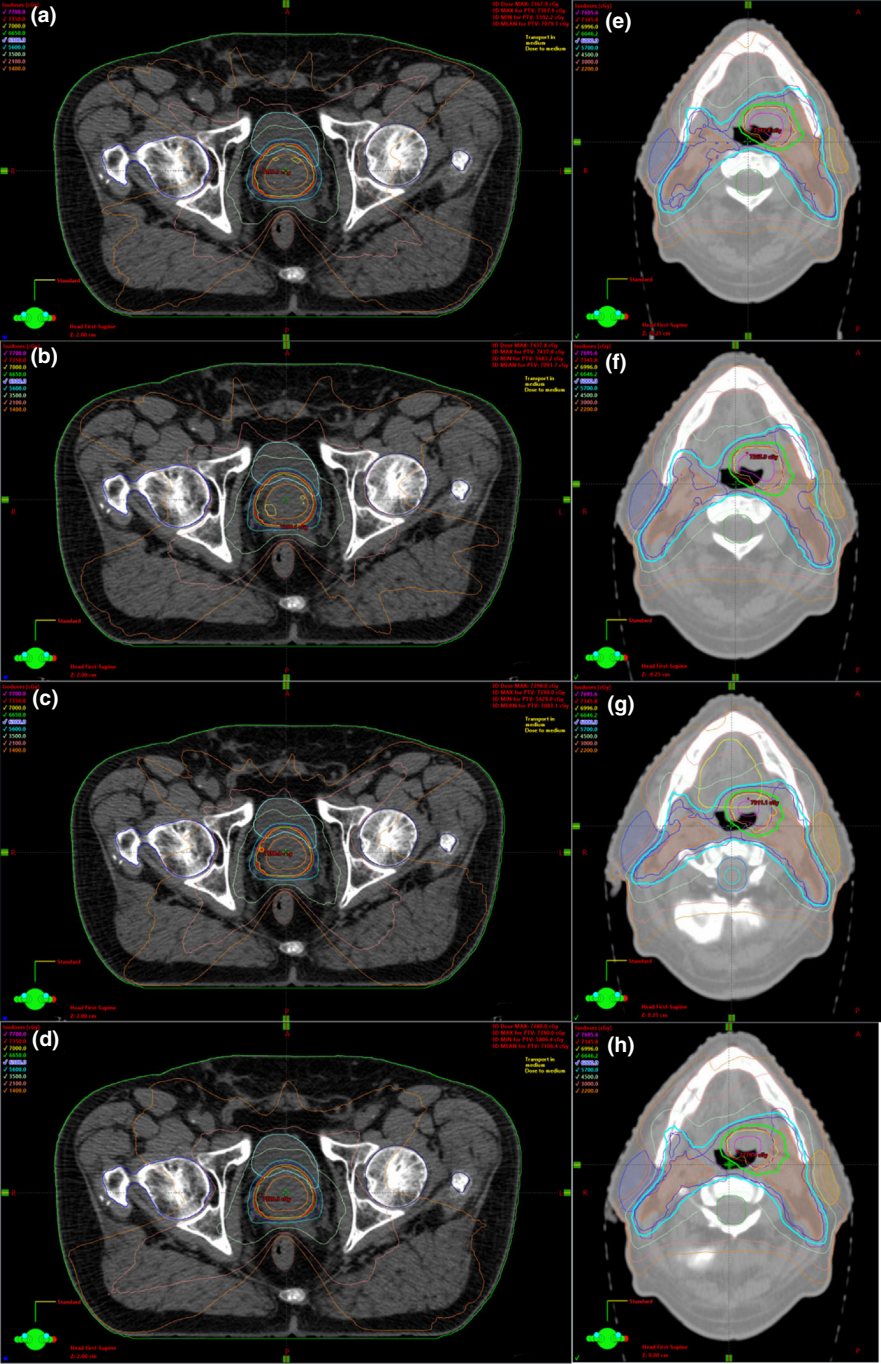
Example of a prostate and an oropharynx plan for the different classes of plans. From top to bottom: Clinical, MU limit, ASC, ASC + MU limit.

**Fig. 2:**
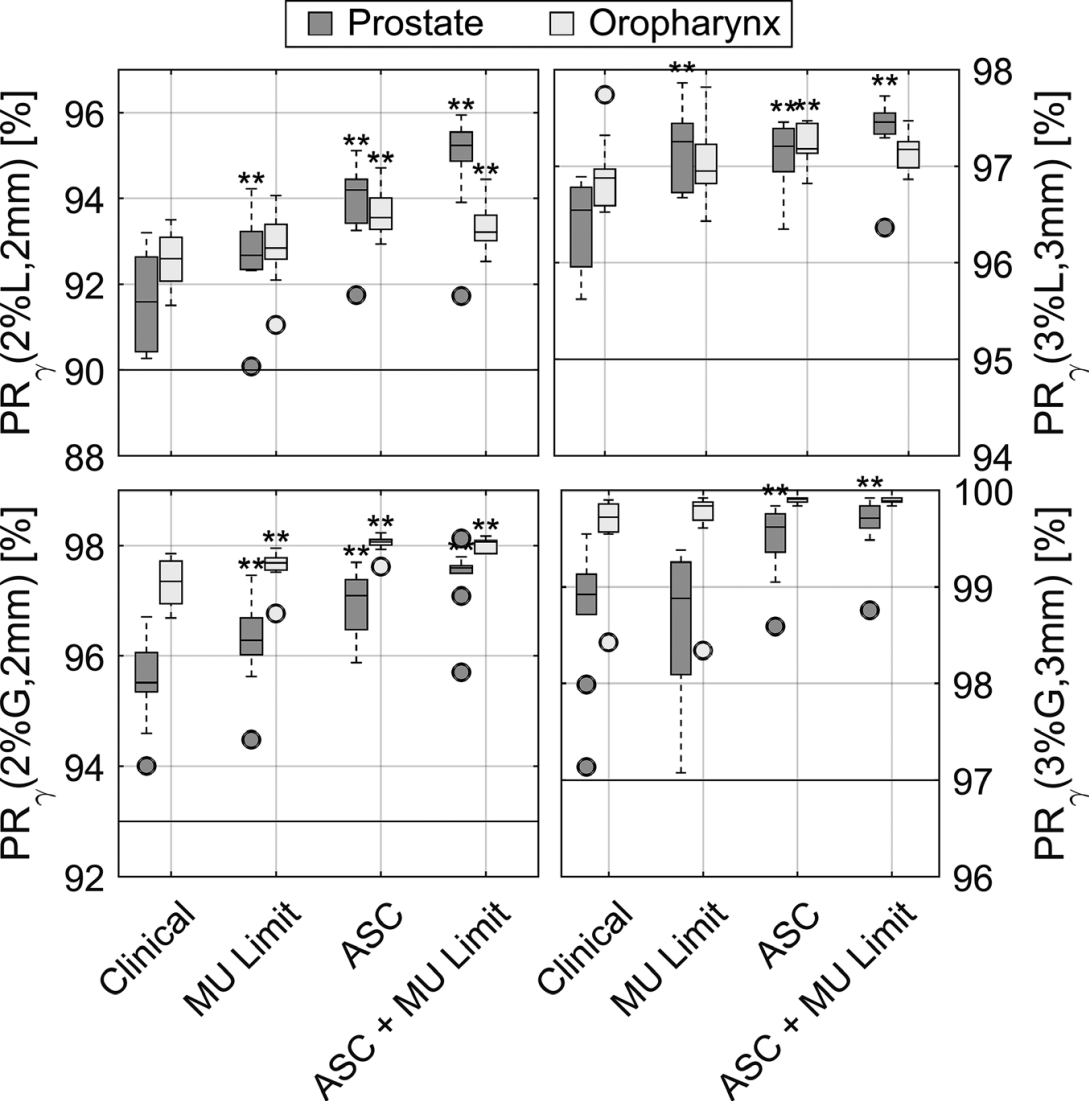
Box and whisker plot of gamma passing rates for the different classes of plans. The central line marks the median, the edges of the box are the 25th and 75th percentiles, the whiskers extend to the adjacent values, which are the most extreme data value that are not outliers, and the circles represent the outliers. Statistically significant differences from clinical plans are marked.

The PQM% of the entire pool of plans is shown in Fig. 1. In general, the overall quality of the clinical plan was maintained by all the other optimization strategies. In Fig. 2 an example of a prostate and an oropharynx plan obtained for the different strategies is given.

Table I depicts the change in dose conformity due to the different optimization strategies. For prostate treatments, a mild improvement of PTV conformity with no significant increase in healthy tissue exposure is seen only for the strategies using the MU limitation. For Oropharynx treatments, the conformity and the healthy tissue doses are generally maintained.

**Table I:** Change in dose conformity.

	**Metric**	**Clinical**	**MU limit**	**ASC**	**ASC + MU limit**
Prostate	CN	0.881 ± 0.021	0.888 ± 0.019*	0.882 ± 0.013	0.895 ± 0.028*
	V66.5Gy [cc]	17.49 ± 4.78	16.75 ± 5.97	17.53 ± 4.27	14.03 ± 4.02*
	V35Gy [cc]	323.3 ± 104.6	321.2 ± 83.4	329.0 ± 142.3	326.9 ± 126.7
	V22Gy [cc]	1147 ± 424	1139 ± 394	1137 ± 484	0.550 ± 0.132
Oropharynx	CN	0.550 ± 0.132	0.548 ± 0.121	0.546 ± 0.082	0.543 ± 0.142
	V56.43Gy [cc]	207.92 ± 51.64	217.19 ± 65.62	219.93 ± 93.90	223.37 ± 128.85
	V66.46Gy [cc]	13.15 ± 8.94	16.12 ± 5.35	14.82 ± 3.08	15.97 ± 5.03
	V35Gy [cc]	1113 ± 196	1127 ± 70	1140 ± 116	1160 ± 104
	V22Gy [cc]	1987 ± 386	1995 ± 265	2000 ± 602	2045 ± 102*

For each metric, the mean ± 1st deviation for each optimization strategy is reported. Significant comparisons are marked with *.

### 3.B Modulation parameters

In the case of prostate treatments, all of the adopted strategies induced significant changes in approximately all the considered modulation parameters, as can be seen in Table II. The number of Monitor Units is reduced together with the overall movement and modulation of the MLC (increase in BEV area and reduction in the number of subfields). Two exceptions are the MI_t_ and the Dose Rate variation: the variation of the former is significant only for *ASC + MU limit* while the latter shows a significant increase for all strategies. It can be concluded that, for all the adopted strategies, the delivery is characterized by a more constant gantry rotation and a reduced MLC travel at the cost of a by a larger modulation of the Dose Rate. Moreover, treatment time is reduced by approximately 10% with a net increase in the size and a net decrease in the complexity of the MLC aperture.

**Table II:** Change in deliverability and modulation parameters for prostate plans.

**Metric**	**Ref. Value**	**Percentage difference**		
	**Clinical**	**MU limit**	**ASC**	**ASC + MU limit**
MU/cGy [cGy−^1^]	5.53 ± 0.81	−27.10 ± 9.76*	−12.46 ± 7.78*	−44.24 ± 7.31*
LTAL [cm/deg]	3.39 ± 0.31	−14.69 ± 7.10*	−10.36 ± 4.12*	−35.67 ± 9.90*
GSV [deg−^1^]	(4.41 ± 1.87) × 10−^2^	−56.69 ± 22.35*	0.48 ± 60.23	−75.08 ± 14.50*
DRV [MU/min/deg]	6.53 ± 1.12	41.21 ± 40.08*	51.12 ± 27.74*	66.31 ± 58.75*
Delivery Time [s]	159.6 ± 13.8	−10.38 ± 7.67*	−3.92 ± 4.13*	−11.03 ± 7.66*
A [cm^2^]	5.18 ± 0.77	32.79 ± 14.53*	12.94 ± 11.22*	71.92 ± 20.17*
EFS [cm]	27.64 ± 2.36	25.93 ± 11.63*	9.73 ± 8.96*	52.91 ± 14.63*
SAS_10mm_ [%]	45.10 ± 3.2	−24.22 ± 9.30*	−11.22 ± 9.33*	−49.44 ± 10.40*
EM	(29.06± 3.41) × 10−^2^	−29.04 ± 9.65*	−20.02 ± 9.80*	−54.57 ± 7.16*
PI	0.10 ± 0.01	37.97 ± 16.53*	57.95 ± 19.83*	166.35 ± 49.15*
PM	0.74 ± 0.04	−12.46 ± 5.04*	−4.60 ± 3.54*	−25.25 ± 6.91*
MCS	0.27 ± 0.02	30.49 ± 16.23*	17.32 ± 9.25*	73.71 ± 25.00*
VMCS	0.32 ± 0.03	14.01 ± 7.15*	11.29 ± 7.64*	41.32 ± 12.64*
MI_t_	28.55 ± 4.28	−0.11 ± 6.82	3.75 ± 5.46	−12.27 ± 9.20*

For each parameter the reference value of Clinical plans is reported in the first column. The other columns report the percentage change of each class of plans with respect to the reference. All values are represented as mean ± 1st deviation. Positive difference means an increase with respect to the clinical plan. Significant comparisons are marked with *.

Concerning prostate plans, the MU limit seems to be more effective than the use of ASC, even if set to very high priority. In general, the largest variations are associated with the *ASC + MU limit* plans, showing that the strategies are somehow complementary and their combined use can lead to bigger changes.

The case of oropharynx treatments, reported in Table III, is similar to what is described above. The only differences are that the dose rate variation is not significantly changed and the reduction in delivery time, even if significant, is not clinically relevant. In this case, the ASC seems to be more incisive than the MU limitation, while the coupling of the two is again the more effective strategy to reduce plan complexity, independent of the considered metric.

**Table III:** Change in deliverability and modulation parameters for oropharynx plans.

**Metric**	**Ref. Value**	**Percentage difference**		
	**Clinical**	**MU limit**	**ASC**	**ASC + MU limit**
MU/cGy [cGy^−1^]	3.92 ± 0.26	−13.20 ± 3.68*	−14.57 ± 6.77*	−24.35 ± 4.77*
LTAL [cm/deg]	4.28 ± 0.21	−5.71 ± 2.93*	−10.17 ± 2.97*	−15.65 ± 2.45*
GSV [deg^−1^]	(0.87 ± 0.49) × 10^−2^	−85.10 ± 18.39*	−15.06 ± 52.01	−61.00 ± 78.10*
DRV [MU/min/deg]	8.53 ± 1.40	−9.91 ± 21.54	19.63 ± 34.91	6.17 ± 30.55
Delivery Time [s]	144.1 ± 2.8	−0.98 ± 0.60*	−0.52 ± 1.06	−0.87 ± 1.15*
A [cm^2^]	13.64 ± 1.68	12.01 ± 3.46*	16.30 ± 12.39*	27.82 ± 11.36*
EFS [cm]	44.01 ± 3.64	11.07 ± 3.24*	13.47 ± 10.54*	23.62 ± 9.32*
SAS_10mm_ [%]	31.8 ± 3.6	−15.40 ± 4.70*	−19.22 ± 12.45*	−35.64 ± 7.73*
EM	(20.57 ± 2.07) × 10^−2^	−15.42 ± 4.60*	−34.45 ± 7.91*	−45.05 ± 4.32*
PI	0.05 ± 0.01	15.70 ± 7.04*	128.49 ± 34.92*	159.24 ± 33.69*
PM	0.81 ± 0.02	−3.46 ± 1.22*	−3.24 ± 2.13*	−6.86 ± 1.48*
MCS	0.25 ± 0.02	12.24 ± 6.28*	19.93 ± 8.90*	35.95 ± 8.63*
VMCS	0.25 ± 0.02	14.45 ± 6.24*	26.75 ± 6.92*	39.77 ± 8.44*
MI_t_	64.87 ± 3.71	−5.21 ± 4.35*	−1.37 ± 5.88	−4.99 ± 6.43*

For each parameter, the reference value of Clinical plans is reported in the first column The other columns report the percentage change of each class of plans with respect to the reference. Positive difference means an increase with respect to the clinical plan. All values are represented as mean ± 1st deviation. Significant comparisons are marked with*.

### 3.C Pretreatment QA

**Fig. 3:**
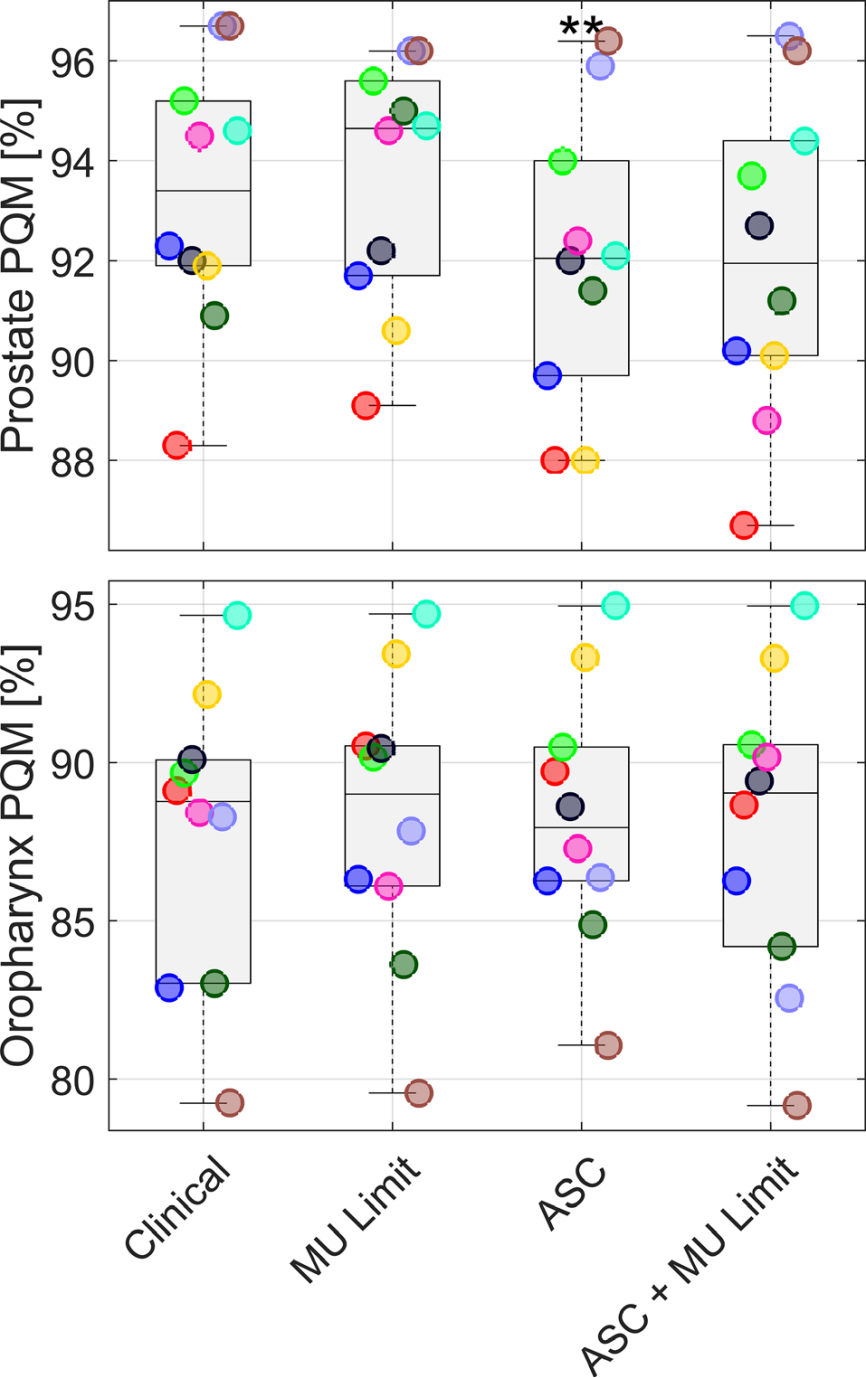
PQM% values for the different optimization strategies. Each color represents a patient. The single data are superposed to box and whisker plot. Top panel refers to prostate plans, bottom panel to oropharynx plans. The central line marks the median, the edges of the box are the 25th and 75th percentiles, the whiskers extend to the adjacent values, which are the most extreme data value that are not outliers, and the circles represent the outliers. Statistically significant differences from clinical plans are marked by **.

Figure 3 clearly shows that the use of ASC increases plan deliverability: GPR% is significantly increased for all considered criteria and for both treatment sites with respect to the clinical plans. GPR% variation induced by the MU limitation is smaller and not as ubiquitous. When the ASC is coupled with the limit on the total MU, the best deliverability is reached for prostate plans while no clear advantage can be seen in the case of oropharynx plans.

## 4 Discussion

The reported results show that prostate and oropharynx plans optimized with PO algorithm v15.5 contain a discrete amount of unnecessary plan complexity that might be mitigated by limiting the total MU or making use of the newly introduced ASC tool during optimization.

Concerning the MU limitation, the results presented here match those already reported in the literature for prostate and hypopharynx, despite the different optimizers.^20^, ^22^

No clinically significant differences in the plan quality was measured for any of the optimization strategies. Not even low dose radiation to normal tissue shows significant variations. A mild but significant decrease only affects *ASC* prostate plans. Nonetheless, large deviations might occur in single cases, as stated by the presence of outliers for *ASC + MU limit* plans. This evidence highlights the need to tailor the ASC penalty to the specific treatment site, performing a detailed analysis on a larger cohort of patients.

For both prostate and oropharynx treatments, a net reduction in the overall plan complexity was observed using either the MU limitation, the ASC set to very high or the coupling of the two. In general, the three strategies lead to plans with a lower number of total MU, a more constant gantry rotation and a MLC modulation characterized by a larger and less complicated shape with leaves traveling shorter overall lengths. For prostate treatments, this is accompanied by 10% reduction in treatment delivery time but larger variations in the instantaneous Dose Rate.

The ASC was less effective in reducing plan complexity in prostate treatments than the MU limitation, the opposite is true for oropharynx treatments and the best results were obtained when the two are coupled. This is probably due to the inherent different anatomical complexity of the two cases which reflects in differently complicated shaping of BEVs. In effect, prostate and SIB oropharynx treatments were chosen as representative of the lowest and highest complexity among RT treatment sites. In other words, in prostate treatments the MLC shaping is inherently simple so that the ASC might have a limited impact. Conversely, in oropharynx treatments the MLC modulation tends naturally toward highly complicated shapes which can be largely simplified by the ASC. It should be noted that the different influences might be due to the particular choice of the 3 MU/cGy limit which implies a stronger limitation for prostate than for oropharynx. It can be seen in Table II and Table III that the reduction in MU/cGy is followed by an increase in the mean aperture area (A), a decrease in its convexity (EM), and to a decrease in the percentage of small aperture (SAS_10 mm_), independent of the applied optimization strategy. This result agrees with the results reported in a large part of the existing literature.^11^, ^13^, ^15^, ^18^, ^19^ Nevertheless, the outcome of the dosimetric verification shows that the ASC grants a better plan deliverability, as measured by all the considered GPR%, than the limit on the total MU. Moreover, for oropharynx treatments, coupling MU limit and ASC does not grant better deliverability even if they are characterized by a lower amount of plan complexity. The results reported herein suggest that the use of ASC and the MU limit might somehow induce complementary changes in the plan complexity.

A partial motivation of this can be found by the positive linear relationship between the uncertainty of 2D pretreatment verification and the proportion of dose from small fields reported in,^9^ but at the moment no clear explanation for such a result can be given because no conclusive relationship has been reported between a single complexity metric and the result of dosimetric verification. A more in‐depth evaluation of the matter is outside the scope of the present work but it is interesting and will be addressed in a subsequent investigation.

This work reports the first experience and the positive impact of ASC on plan complexity and deliverability. The results reported herein suggest the need for further investigation on a larger patient cohort to tweak the ASC setting to the specific treatment site, eventually coupling it to the MU limit.

## 5 Conclusions

The use of ASC in Eclipse optimization leads to a reduction in plan complexity comparable or superior to a 15% reduction in the total delivered monitor unit. On average, the reduced complexity does not compromise the overall clinical plan quality but ensures an ameliorated plan deliverability. Detailed results might vary among different treatment sites. ASC deserves additional studies for a thorough evaluation of its capability and methods of use.

## Conflict of Interest

None.

The author(s) received no specific funding for this work. A. Scaggion and M. Fusella were responsible for statistical analyses of the entire work.

## Supporting information

Fig S1. Metrics related scoring functions that compose the PQM algorithm used to assess prostate plan quality.
Fig S2. Metrics related scoring functions that compose the PQM algorithm used to assess oropharynx plan quality.
