# Supplementary material for: Limiting treatment plan complexity by applying a novel commercial tool
Source: J Appl Clin Med Phys. 2020 May 21;21(8):27–34. doi: 10.1002/acm2.12908 (PMC7484888; doi:10.1002/acm2.12908)
Supplement: Supplementary file 1 — Fig S1. Metrics related scoring functions that compose the PQM algorithm used to assess prostate plan quality. Fig S2. Metrics related scoring functions that compose the PQM algorithm used to assess oropharynx plan quality. [file ACM2-21-27-s001.docx]

# **Supplementary Material**


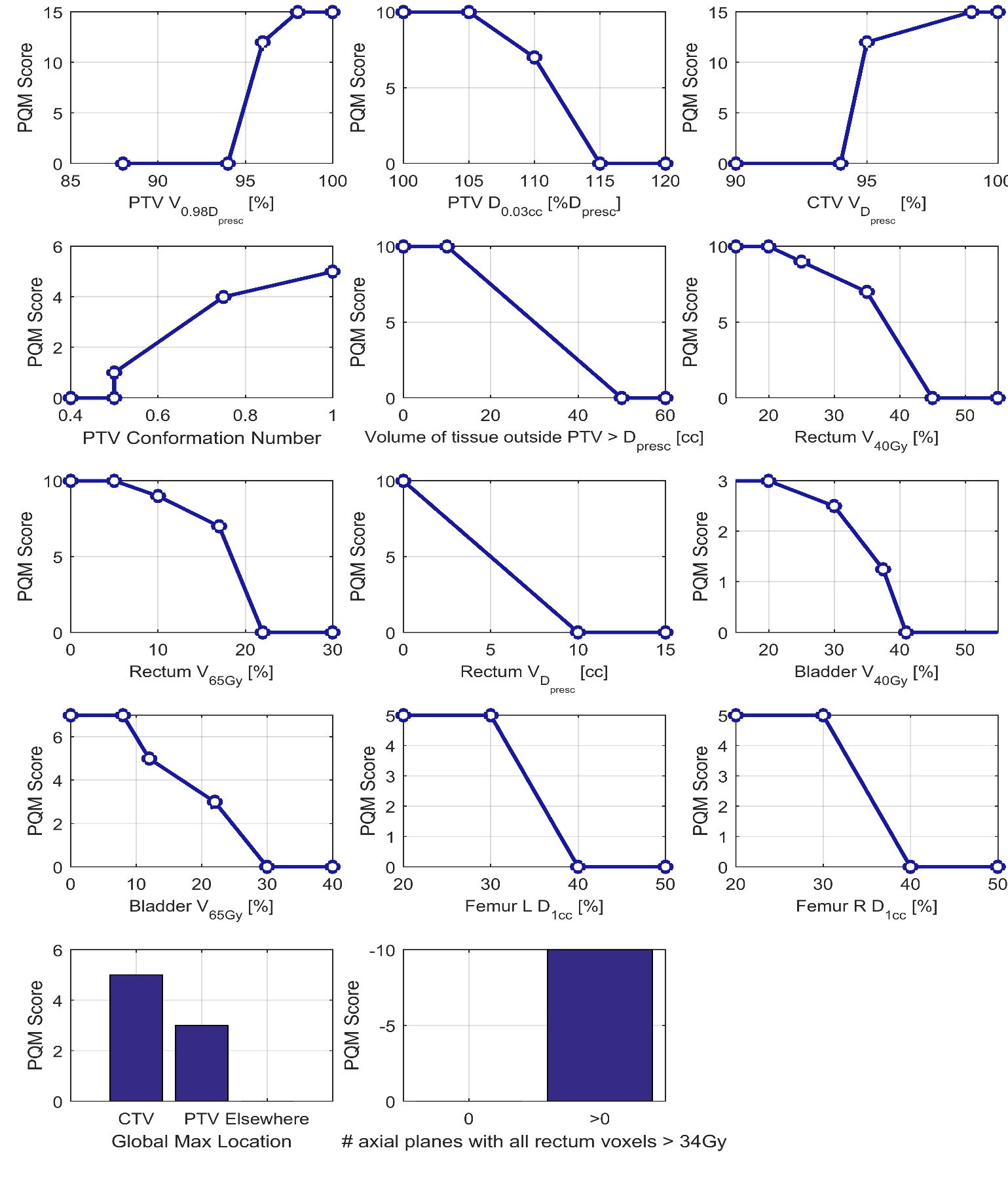


Figure 1 Metrics related scoring functions that compose the PQM algorithm used to assess prostate plan quality.


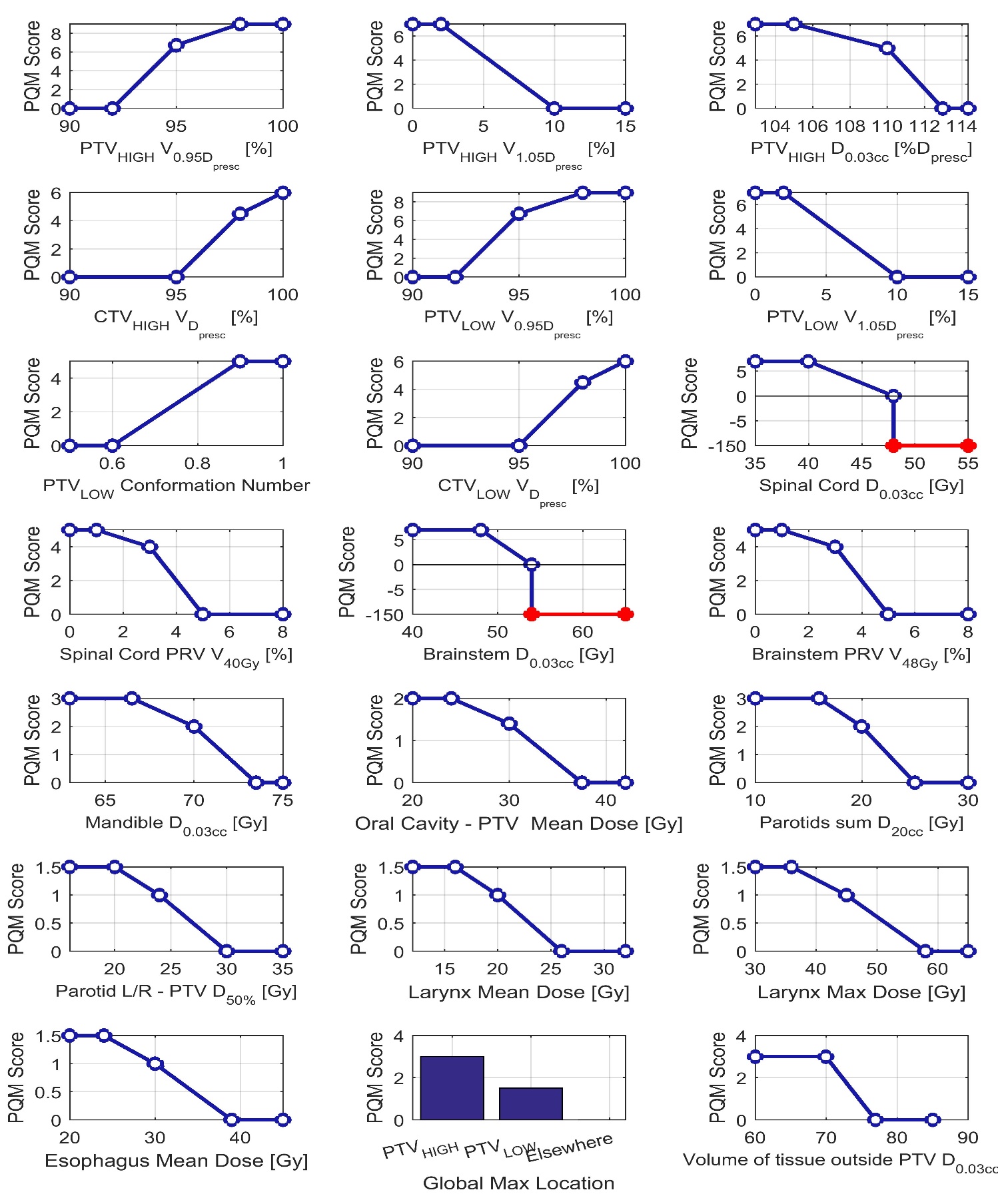


Figure 2 Metrics related scoring functions that compose the PQM algorithm used to assess oropharynx plan quality.
